# Temporal binding of sound emerges out of anatomical structure and synaptic dynamics of auditory cortex

**DOI:** 10.3389/fncom.2013.00152

**Published:** 2013-11-07

**Authors:** Patrick J. C. May, Hannu Tiitinen

**Affiliations:** Brain and Mind Laboratory, Department of Biomedical Engineering and Computational Science, School of Science, Aalto University, Aalto, Finland

**Keywords:** adaptation, auditory cortex, combination sensitivity, computational model, inhibition, stimulus selectivity, synaptic depression, temporal binding

## Abstract

The ability to represent and recognize naturally occuring sounds such as speech depends not only on spectral analysis carried out by the subcortical auditory system but also on the ability of the cortex to bind spectral information over time. In primates, these temporal binding processes are mirrored as selective responsiveness of neurons to species-specific vocalizations. Here, we used computational modeling of auditory cortex to investigate how selectivity to spectrally and temporally complex stimuli is achieved. A set of 208 microcolumns were arranged in a serial core-belt-parabelt structure documented in both humans and animals. Stimulus material comprised multiple consonant-vowel (CV) pseudowords. Selectivity to the spectral structure of the sounds was commonly found in all regions of the model (*N* = 122 columns out of 208), and this selectivity was only weakly affected by manipulating the structure and dynamics of the model. In contrast, temporal binding was rarer (*N* = 39), found mostly in the belt and parabelt regions. Thus, the serial core-belt-parabelt structure of auditory cortex is necessary for temporal binding. Further, adaptation due to synaptic depression—rendering the cortical network malleable by stimulus history—was crucial for the emergence of neurons sensitive to the temporal structure of the stimuli. Both spectral selectivity and temporal binding required that a sufficient proportion of the columns interacted in an inhibitory manner. The model and its structural modifications had a small-world structure (i.e., columns formed clusters and were within short node-to-node distances from each other). However, simulations showed that a small-world structure is not a necessary condition for spectral selectivity and temporal binding to emerge. In summary, this study suggests that temporal binding arises out of (1) the serial structure typical to the auditory cortex, (2) synaptic adaptation, and (3) inhibitory interactions between microcolumns.

## Introduction

The essence of natural sounds lies in their temporal structure. For example, speech sounds have a rich spectral mix of harmonic components, band-pass noise, and silent periods (Fant, 1970; Kent and Read, 1992). The temporal order in which these components are delivered determines whether vocalizations amount to intelligible speech and, ultimately, what their semantic interpretation is (Marslen-Wilson and Welsh, 1978; Klatt, 1979). Other examples of sounds whose temporal structure lends them a meaningful interpretation include music and animal communication sounds. To process such sounds, the brain must therefore rely not only on spectral analysis, but also on temporal binding of spectral information over varying time spans. It seems that the cochlea, in the initial part of the auditory pathway, provides a filter bank which feeds information into multiple tonotopic streams of the subcortical auditory system; these in turn provide auditory cortex with a representation of the distribution of sound energy across frequency which is robust against intensity changes (for a review, see Young, 2008). Spectral analysis would therefore seem to be a redundant task for auditory cortex, and it has been suggested that its function is to perform temporal binding (Nelken, 2004). In indirect support of this, there is a wealth of observations from cortex confirming the fact that nerve cells respond selectively to temporally complex sounds (McKenna et al., 1989; Rauschecker et al., 1995; Wang et al., 1995; Brosch and Schreiner, 1997, 2000; Rauschecker, 1997; Brosch et al., 1999; Tian et al., 2001; Kilgard and Merzenich, 2002; Bartlett and Wang, 2005; Brosch and Scheich, 2008; Recanzone, 2008; Sadagopan and Wang, 2009). Behaviorally, temporal binding appears to operate concurrently on many time scales, as is made evident by our ability to make sense of speech both on the word and sentence levels. What remains unclear are several central aspects of the neural basis of temporal binding: What determines the time span of binding? How is the variety of time scales of binding achieved? How does learning and memory interplay with temporal binding? How does temporal binding in the auditory modality differ from that in the visual modality? However, before we can even begin to answer these questions—either experimentally of through computational modeling—we must first address the very basic issue of *what* temporal binding is in terms of brain activity.

An intracortical window into the structure of the auditory cortex is provided by the results from primates (Pandya, 1995; Hackett et al., 1998; Kaas and Hackett, 2000): Subcortical information originating from the cochlea arrives via the inferior colliculus and the thalamus to at least three core areas of auditory cortex (including primary auditory cortex) in the lateral sulcus, each containing sharply-tuned, tonotopically organized cells. The core areas are surrounded by eight belt areas comprising several tonotopic fields. Belt areas receive feedforward input from the core and are also modulated by reciprocal connections between neighboring belt areas. In turn, belt areas are bordered by lateral parabelt areas, which lack direct input from the core and are therefore driven specifically by the belt. Thus, feedforward activation in auditory cortex is sequential due to the core-belt-parabelt progression of activity. However, this feedforward connectivity is complemented by feedback connections which progress in reverse order, from the parabelt to the belt, and from the belt to the core. Further, as areas in each stage are interconnected most densely with their nearest neighbors, several parallel core-belt-parabelt streams are formed, with an overall rostral-caudal subdivision being evident. A similar organization has been identified in humans: The primary auditory cortex, in the postero-medial part of Heschl's gyrus (HG), is made up of three tonotopically organized fields and is surrounded by belt areas on the lateral part of HG and along the planum temporale (PT) and the superior temporal gyrus (STG; Galaburda and Sanides, 1980; Rivier and Clarke, 1997; Sweet et al., 2005). Serial activation, consistent with a core-belt-parabelt structure, is evident in intracortical (Yvert et al., 2005; Guéguin et al., 2007; Gourévitch et al., 2008) and non-invasive (Inui et al., 2006; Chevillet et al., 2011) measurements in human auditory cortex. Thus, the auditory cortex of both humans and non-human primates seems to be uniquely characterized by multiple parallel streams of information flow with a distinctly serial, three-level structure. This can be contrasted with the visual and somatosensory cortices, where primary areas not only connect to immediately surrounding belt areas but also bypass these by connecting directly with anatomically more distant areas (see Kaas and Hackett, 2000).

Auditory cortex is characterized by adaptation, the short-term modification of the responsiveness of neurons by auditory stimulation, which is mostly suppressive in nature and lasts up to seconds. This phenomenon—also known as forward suppression and forward masking—seems to be stimulus-specific and can be observed both intracortically and non-invasively. In single-cell recordings in the cat primary auditory cortex, the response to a probe tone can be diminished if it is preceded by a masker tone (Calford and Semple, 1995; Brosch and Schreiner, 1997, 2000; Ulanovsky et al., 2003). When the probe and the masker tone are both set to the characteristic frequency of the cell, the magnitude of this forward suppression effect as well as the recovery time from it (53–430 ms) are maximized. Similar probe-masker effects can be observed in MEG and EEG measurements in humans: The most prominent response in the auditory event-related potential is the N1 and its magnetic counterpart N1m (also known as N100 and N100m). The N1(m) peaks at round 100 ms after stimulus onset and, compared to other event-related responses, is a particularly sensitive indicator of adaptation: the magnitude of the N1(m) is diminished already after a single stimulus repetition, and the recovery from this adaptation takes several seconds (for a review, see May and Tiitinen, 2010). Adaptation observed in cortex seems to be cortical in origin rather than being an effect which is produced subcortically and merely passed on to the response patterns of cortical neurons. (Brosch and Schreiner, 1997; Ulanovsky et al., 2004; Wehr and Zador, 2005; see also Calford and Semple, 1995). Synaptic depression is the most likely candidate for the mechanism of adaptation (Wehr and Zador, 2003, 2005). This conclusion is supported by measurements on the time scales of adaptation (Ulanovsky et al., 2004): Stimulus-specific adaptation has several concurrent time scales, ranging from a few milliseconds to tens of seconds, which seem to reflect those present in the stimulation. Importantly, these time scales match the several co-existing time constants which describe the lifetime of synaptic depression of corticocortical synapses (Tsodyks and Markram, 1997; Varela et al., 1997; Markram et al., 1998). Further, computational modeling studies show that synaptic depression alone can account for the response patterns associated with stimulus-specific adaptation (Mill et al., 2011, 2012).

Temporal binding performed by auditory cortex is indicated by intracortical results. Cells in core and belt areas of auditory cortex exhibit enhanced responses to sounds when these are presented as part of sound sequences rather than as isolated stimuli, as has been found in the case of pure tones (McKenna et al., 1989; Brosch and Schreiner, 1997, 2000; Brosch et al., 1999; Brosch and Scheich, 2008; Sadagopan and Wang, 2009) noise sequences, (Kilgard and Merzenich, 2002), and amplitude-modulated sounds (Bartlett and Wang, 2005). These cases demonstrate temporal combination sensitivity (CS), that is, an auditory stimulus elicits a weak response when it is presented in isolation but a strong response when it is immediately preceded by a specific sequence of sound. Temporal binding is also required by core and belt neurons which show selectivity to the direction of frequency modulation (e.g., Tian and Rauschecker, 1994, 1998, 2004; Kowalski et al., 1995; Godey et al., 2005). Responses to species-specific vocalizations also point to spectral and temporal binding. Single-cell measurements in rhesus monkey reveal that cells in the belt and parabelt respond selectively to monkey calls (Rauschecker et al., 1995) and also show temporal CS (Rauschecker, 1997). Also, cells which respond preferentially to monkey calls compared to their time-reversed versions can be found in the marmoset (Wang et al., 1995) and macaque (Recanzone, 2008). In the rhesus monkey, a preponderance of cells selective to call identity has been found in a pathway that extends from the lateral belt areas anterior to the core, and extends to anterior prefrontal cortex (Tian et al., 2001). This kind of selectivity might, hypothetically, tie in with non-invasive results from the human brain on selectivity to the acoustic-phonetic content of speech content (Leaver and Rauschecker, 2010). Also, results from the human brain indicate the presence of areas in the anterior temporal cortex which are selectively activated by speech (Binder et al., 2000), its acoustic-phonetic content (Leaver and Rauschecker, 2010; DeWitt and Rauschecker, 2012), and melodic structures (Patterson et al., 2002).

However, the neural mechanisms of temporal binding are currently unknown. Delay lines have been suggested as a solution, whereby a vocalization-selective cell is activated by the concurrent arrivals of delayed and on-time representations of the respective initial and later portions of the vocalization content (Leaver and Rauschecker, 2010). Similar delay mechanisms have also been suggested as an explanation of FM response selectivity (e.g., Voytenko and Galazyuk, 2007; Ye et al., 2010) and of the autocorrelation analysis underlying pitch perception (e.g., Licklider, 1951; Meddis and Hewitt, 1991). Given the time span of human and monkey vocalizations, this explanation would require cortical activation delay lines of (at least) several hundred milliseconds. As this is physiologically somewhat implausible in view of the 10-ms delay between cochlea and cortex activations (Liégeois-Chauvel et al., 1994), it may be prudent to investigate other possibilities. We recently suggested that the mechanism of temporal binding in auditory cortex is provided by stimulus-specific adaptation expressed through activity-dependent depression of synaptic strengths on the single-cell level and modifications of the N1(m) response on the mass-action level (May and Tiitinen, 2007, 2010). In this scheme, each stimulus modifies the auditory cortex so that subsequent stimuli are processed in a system which bears the memory traces of past events. Expressed in terms of artificial neural networks, the activation of the network modifies the network weights in a local manner. Consequently, the structure of the network evolves with the stimulation and, thus, the input–output transformation becomes dependent on the set of past input patterns and their temporal order. A related principle has been suggested to underlie pitch perception: the cortical activity elicited by incoming stimuli uses top-down connections to modulate the dynamics of subcortical areas; this results in a temporal window of integration which adapts according to current stimulation and allows for pitch perception to occur across a wide range of stimulus periodicities (Balaguer-Ballester et al., 2009).

Here, we explore how the neural mechanisms of temporal integration might be explained by the hierarchical structure of auditory cortex combined with synaptic depression. We modeled auditory cortex as a system comprising multiple core, belt, and parabelt areas. Further, the structure of auditory cortex was approximated through (1) topographic connectivity between areas, (2) feedback connections from parabelt to belt and from belt to core, (3) a presence of multiple parallel core-belt-parabelt streams, and (4) a serial core-belt-parabelt structure. In simulations of the model, we varied the structure of the model, the decay time of synaptic depression, and the proportion of inter-column inhibitory connections in an attempt to capture the features which are important for the emergence of cortical sensitivity to the spectral and temporal structure of complex, naturally-occurring sounds. As stimulus material, the simulations used pseudowords comprising random combinations of consonant-vowel (CV) pairs. The core, belt, and parabelt regions of the model were probed for the ability of their constituent neural populations to respond selectively to the stimuli, that is, to differentiate the stimuli in terms of the amplitude of stimulus-elicited activity. Importantly, we tested how well these regions performed temporal binding of the stimuli. This was achieved by presenting CV components of the stimuli in isolation as well as time-reversed versions of the stimuli: a stronger response to the original, intact stimulus compared to these modifications (which retained the original spectral structure) indicated temporal binding ability. We hypothesized that synaptic depression would be crucial for temporal binding. Also, we expected that the parallel and serial connectivity pattern of auditory cortex would be useful for temporal binding.

## Methods

### Model dynamics and structure

#### Dynamics

**Figure 1:**
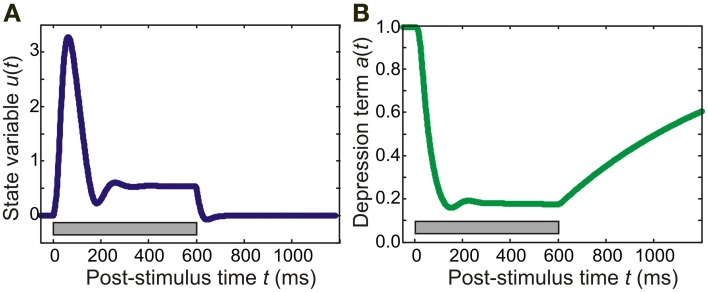
**Time course of model variables.** In this example, the model was presented with a 600-ms pure tone of frequency 5700 Hz. **(A)** The panel shows the behavior of an excitatory population in the core area for which the pure tone is the preferred stimulus. The state variable *u* exhibits a transient response peaking at around 50 ms. This response is then followed by sustained activity which rapidly dies away after stimulus offset. **(B)** The adaptation (suppression) term of a synaptic connection made by the excitatory population starts out at 1.0, the resting value. After stimulus onset, it reaches a minimum of around 0.2 at around 100 ms; after stimulus offset, adaptation recovers slowly (taking several seconds to reach the resting value again). The gray bar indicates stimulus duration.

We simulated auditory cortex with a model comprising *N* = 208 “microcolumns,” each containing a population of excitatory (pyramidal) cells and a population of inhibitory interneurons. The basic unit of the model was the pooled activity of such an excitatory or inhibitory population described through the Wilson and Cowan firing rate model (Wilson and Cowan, 1972). Thus, for each population, firing rate *g* depended on the state variable *u* through a non-linear monotonically increasing function *g*(*u*) = tanh (2/3) (*u*−θ) when *u* > θ, *g*(*u*) = 0 otherwise, where θ = 0.1 is a threshold constant. With the state variables of the excitatory and inhibitory cell populations described by the vectors **u** = [*u*_1_ … *u*_*N*_] and **v** = [*v*_1_ … *v*_*N*_], respectively, the dynamic equations describing neural interactions are

(1) $$\begin{array}{l} \tau_{m}\dot{u}(t)=-u(t)+W_{\text{ee}}\cdot g\text{​}\left[u(t)\right]-W_{\text{ei}}\cdot g\text{​}\left[v_{i}(t)\right]+I_{\text{aff}}(t),\\ \;\tau_{m}\dot{v}(t)=-v(t)+W_{\text{ie}}\cdot g\text{​}\left[u(t)\right] \end{array}$$

where τ_m_ = 30 ms is the membrane time constant, *W*_ee_ > 0 is the matrix of excitatory synaptic weights connecting the pyramidal populations to each other, *W*_ie_ > 0 represents the weights from pyramidal populations to interneuron populations, and *W*_ei_ > 0 are the weights from the interneurons to the pyramidal cells. Further, **I**_aff_ is the vector describing afferent input arriving to cortex from the auditory pathway. Synaptic depression was assumed to affect the interactions between the pyramidal cells and was realized by modifying *W*_ee_ by a time-dependent depression term *a*(*t*) so that the effective synaptic weight between columns *i* and *j* is *a*_*ij*_(*t*)*w*_*ij*_ and depends on the presynaptic activity through

(2) $${\dot{a}}_{ij}(t)=\frac{1-a_{ij}(t)}{\tau_{\text{a}}}-ka_{ij}(t)g\left[u_{j}(t)\right],$$

where τ_a_ = 0.8 s is the time constant of adaptation and *k* = 20 is a constant (for similar models of auditory cortex, see May et al., 1999; Loebel et al., 2007; May and Tiitinen, 2010). An example of the time course of adaptation is shown in Figure 1B: In this instance, continuous pure-tone stimulation results in a relatively fast onset of adaptation, with *a*(*t*) reaching a minimum within around 100 ms. Recovery after stimulation was slower, with *a*(*t*) taking several seconds to approach its resting state of *a* = 1.

#### Structure

**Figure 2:**
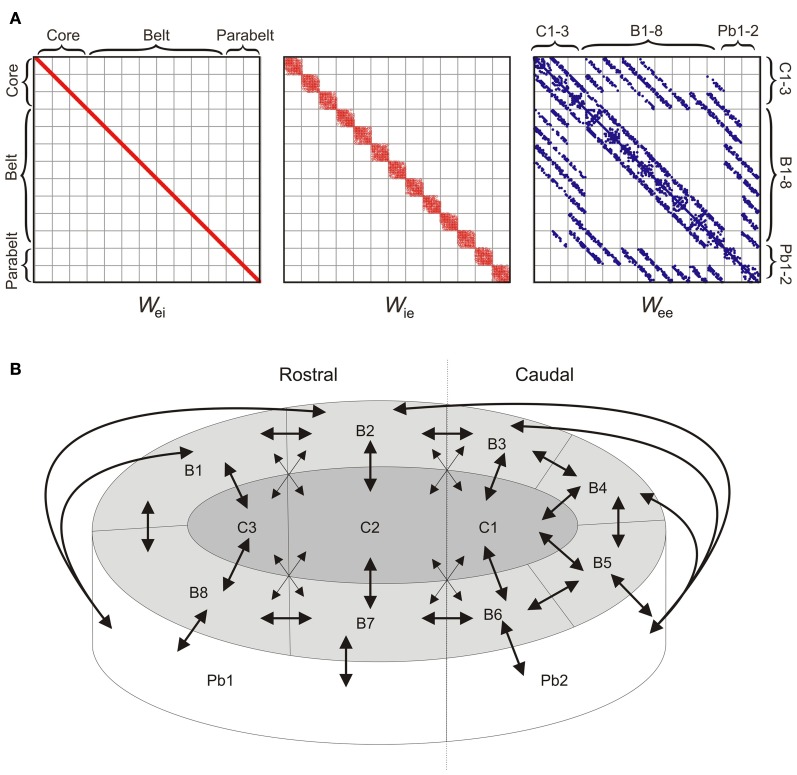
**The model structure. (A)** The 208 × 208 weight matrices *W*_ei_, *W*_ie_, and *W*_ee_ of the model determine the intra- and inter-area connectivity between the columns which were divided into 13 areas: three core areas (C1-3), eight belt areas (B1-8), and two parabelt areas (Pb1-2). Afferent input (not shown) targets the three core areas. Inhibition is local in the sense that the inhibitory interneuron population of each column targets the population of excitatory neurons of the same column. Thus, *W*_ei_ has non-zero values along the diagonal only. Inhibition is local also in the sense that the interneuron population receives excitatory input from within the same area only, and hence *W*_ie_ has a diagonal structure. Connections between excitatory populations occur within the column (diagonal values of *W*_ee_), within each area (diagonal subdivisions of *W*_ee_), and between areas (off-diagonal subdivisions of *W*_ee_). Because of topographic connectivity, each subdivision of *W*_ee_ has a diagonal structure. Connections below and above the diagonal subdivisions are feedforward and feedback connections, respectively. Dots represent non-zero values. **(B)** A schematic diagram of the model (equivalent to *W*_ee_ above) shows the inter-area connectivity of the model. Mimicking auditory cortex, the network consisted of core (C1-3), belt (B1-8), and parabelt (Pb1-2) regions, each divided into multiple areas with denser and sparser interconnections (denoted by large and small arrowheads, respectively). With afferent input targeting the core areas only, there were multiple streams of feedforward activation, all of them progressing serially from the core to the belt and from the belt to the parabelt. Connections between areas were topographic and bi-directional.

The 208 columns were divided into 13 cortical areas, each containing *N*_*F*_ = 16 columns, where each column comprised one excitatory and one inhibitory population (as described above). The structure of the model was determined at three levels of resolution: connections within a column, connections within an area, and connections between areas. Synaptic weights were strongest within a column, with intra-column recurrent excitation mediated through diagonal values of *W*_ee_ set to *w*_jj_ = 6. Inhibition was assumed to be local, with the interneurons of each column projecting only to the pyramidal cells of that column. Thus, as shown in Figure 2A, the only non-zero values of *W*_ei_ were on the diagonal and had a magnitude of 10. The local, interneuron-targeting excitatory connection within the column had a magnitude of 2 (i.e., the diagonal values of *W*_ie_). As shown in Figure 1A, these parameter values resulted in columns in the core region responding to preferred pure tone stimulation with a transient response followed by sustained activity of stimulus duration (see Wang et al., 2005).

Within each area, inter-column connections originated from the pyramidal cells though *W*_ee_ and *W*_ie_. In each of these matrices, these intra-area connections were described by the 13 subdivisions along the diagonal, where each subdivision can be thought of as constituting a 16 × 16 “intra-area” matrix (Figure 2A). The probability of a directed connection between two columns was *p*_0_ = 0.75. Connection probability had a Gaussian drop-off from the diagonal with a standard deviation of σ = 0.6 *p*_0_*N*_*F*_ (Levy and Reyes, 2012). These excitatory connections could either be functionally excitatory (via *W*_ee_) or functionally inhibitory (via *W*_ie_), that is, they targeted the pyramidal or interneuron population of the receiving columns, respectively. Note that in the following, *functionally inhibitory* connections refer to the excitatory connections made from one column to the inhibitory interneuron population of another column. Evidence for such lateral inhibitory interactions has been found in A1 (Kurt et al., 2008; Moeller et al., 2010), and was assumed to hold for all areas in the model. These excitatory (*W*_ee_) and interneuron-targeting, functionally inhibitory (*W*_ie_) lateral connections had a magnitude of 0.5 and 2.0, respectively. The probability that an intra-area connection was functionally inhibitory rather than excitatory was *p*_inh_ = 0.8. All inhibition in the model was local in the sense that inhibitory populations projected within the column (i.e., *W*_ei_ had diagonal values only), and were targeted by pyramidal populations from within the same area (i.e., the only non-zero values of *W*_ie_ were in the 13 subdivisions along the diagonal). Thus, with the assumption that all inter-area connections were functionally excitatory, this resulted in an overall proportion of 50% inhibitory connections in the entire model. Simulations, not shown here, demonstrated that allowing for global inhibitory connections between areas had no effect on model performance.

Inter-area connectivity was modeled on results from primates (Hackett et al., 1998). Connections between areas were assumed to be functionally excitatory and thus were described through off-diagonal values of *W*_ee_. Afferent tonotopically organized input **I**_aff_ targeted three interconnected “core” areas only. These were interconnected with eight surrounding “belt” areas which, in turn, were interconnected with two “parabelt” areas (Figure 2B). With no direct connections between the core and parabelt, the model had a serial structure. Strong and weak connectivity as indicated by the results of Hackett et al. (1998) was equivalent to connection probabilities *p*_1_ = 0.1 and *p*_2_ = 0.05, respectively. With core and belt connections occurring only between neighboring areas in the layout illustrated in Figure 2B, this resulted in multiple core-belt-parabelt streams of connections with a roughly “rostral” and “caudal” subdivision. Connections between areas were topographic (De la Mothe et al., 2006) with most connections occurring near the diagonal on the relevant subdivision of *W*_ee_ (Gaussian drop-off, σ = 0.6 *pN*_F_). Thus, as shown in Figure 2A, each inter-area subdivision of *W*_ee_ was characterized by a diagonal structure. Assuming a 10-ms signal delay from cochlea to cortex (Liégeois-Chauvel et al., 1991), the above setup resulted in onset latencies of 17, 39, and 54 ms for the core, belt, and parabelt region, respectively (measured through *g* of the excitatory population of the microcolumn which generates the maximal response in each region). These onset latencies agree well with non-invasive results from the human auditory cortex (serial activation 17–48 ms; Inui et al., 2006; see also May and Tiitinen, 2010).

### Stimuli

**Figure 3:**
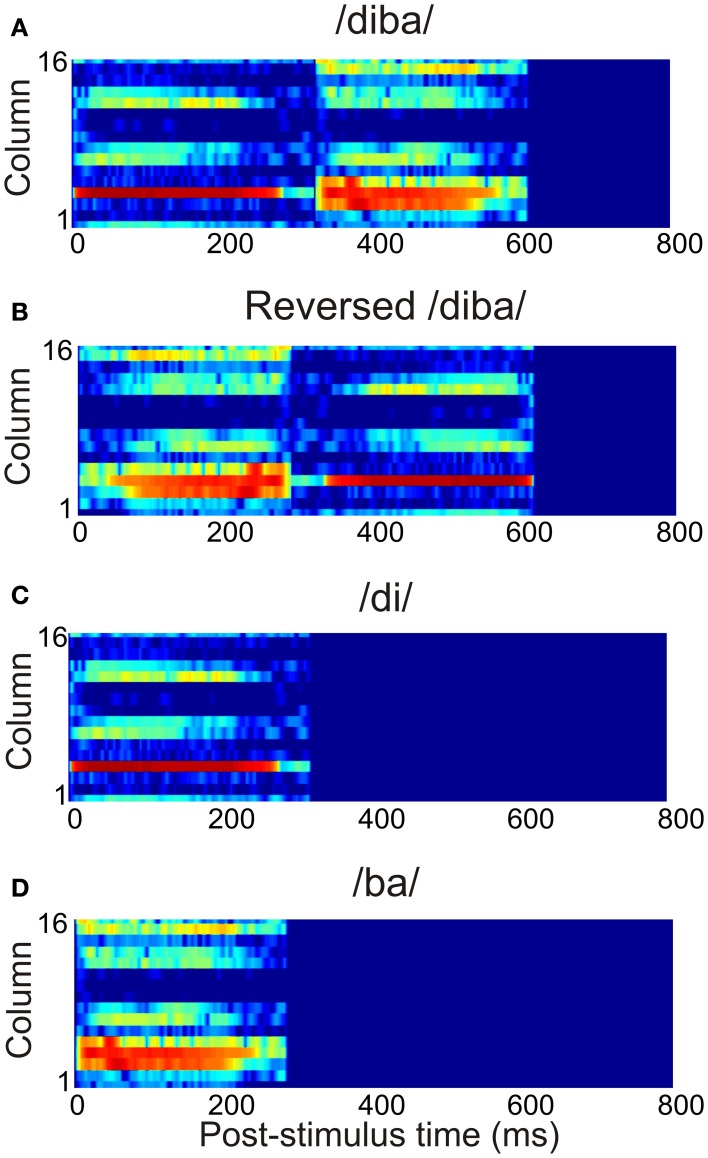
**The time-frequency representation of the afferent stimulation targeting each core area.** This example shows the activation due to **(A)** the pseudoword /diba/, **(B)** its time reversed version, and **(C,D)** the separate CV stimuli /di/ and /ba/.

The current study used 12 American-English CV combinations of voiced stop consonants /b/, /d/, and /g/ and vowels /a/, /ae/, /i/, and /u/ (Stephens and Holt, 2011). These were randomly combined into eight CVCV pseudowords with an average duration of 663 ms (range: 570–712 ms). The stimuli were normalized with respect to their root-mean-square values. To model the spectral analysis carried out by the subcortical auditory pathway, the stimuli were transformed into spectrograms with 16 frequency channels (spanning frequencies 20–13600 Hz) and 1-ms time resolution. This crude approximation provided a tonotopic representation of the stimuli capturing the time-evolution of their spectral content. An example of the time-frequency representation of a pseudoword is shown in Figure 3A. To study the sensitivity of the model to the temporal structure of stimulation, the stimuli were also presented as time-reversed versions (Figure 3B). Also, the pseudowords were divided into their constituent CV stimuli and these were presented separately to the model (Figures 3C,D). Thus, the total stimulus set comprised 28 vocalizations. All stimuli had a linear onset and offset ramp of 5 ms. The stimulus spectrograms were normalized to unity and presented to the three core areas, with each frequency channel targeting one column per core area.

### Analysis

To study the selectivity of the model to the speech stimuli, we calculated the Preference Index (PI) for each column on the basis of the firing rate *g* of the pyramidal cell population. The PI, utilized in primate studies to measure selectivity to species-specific vocalizations (e.g., Rauschecker et al., 1995; Tian et al., 2001; Recanzone, 2008), is derived by first identifying the preferred stimulus (PS), that is, the stimulus that elicited the maximal response. The PI is then defined as the number of stimuli to which a neuron yielded a response whose maximum amplitude was at least 50% of the maximal response. With our set of eight stimuli, an index value of 1–3 indicated “strong” preference, that is, the column responds selectively to only a small subset of the stimuli; an index of 4 and above indicated “weak” preference, where the column's ability to distinguish between stimuli is low (see Rauschecker et al., 1995). A column showing strong preference to a subset of the stimuli is evidence for spectral selectivity, but this does not yet entail sensitivity to temporal structure.

Temporal binding in a column was demonstrated when the column exhibited temporal CS. To meet the requirements of CS, a column had to produce a large response, in terms of the maximal value of the firing rate *g* of the pyramidal cell population, only when the spectral content of the PS was delivered in a specific order (Rauschecker, 1997) or when the PS was presented in its original form as opposed to its time-reversed version (Wang et al., 1995). Accordingly, we used the time-reversed pseudowords and the constituent CV syllables to measure whether the columns of the model exhibited CS. A column was considered temporally sensitive if the magnitude of the response (i.e., the maximal value of *g*) to the PS was more than double that to the reversed version of the PS and to the constituent CV elements presented in isolation.

**Figure 4:**
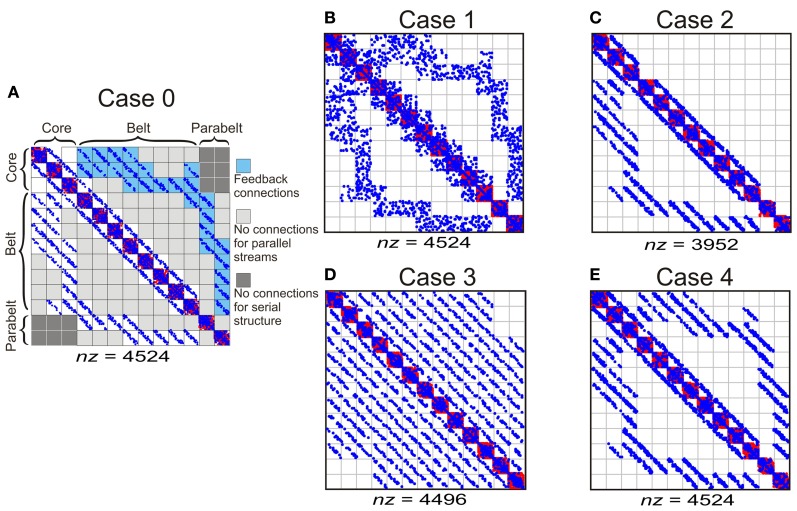
**The column-to-column connection matrices of the intact model (Case 0) and of the structural modifications of the model (Cases 1–4).** The columns were divided into 13 areas designated into core, belt, and parabelt regions. **(A)** Case 0 represents the intact model and represents the connectivity shown schematically in Figure 2. The parallel structure of the model comes about by the connections targeting nearest neighbor areas (i.e., no connections in light gray sections). The serial structure arises out of afferent input targeting the core areas only (not shown) and by the lack of core-parabelt interconnections (dark gray). Blue and red dots signify functionally excitatory (*W*_ee_) and inhibitory connections (*W*_ie_), respectively. The total number of connections is represented by *nz*. **(B)** In Case 1, the topographic connections from one area to another have been replaced by random connections. **(C)** In Case 2, the belt-core and parabelt-belt feedback connections have been removed. **(D)** In Case 3, the parallel structure has been abolished by redistributing the connections so that areas are interconnected equally densely irrespective of inter-area distance. The serial structure is maintained in this modification. **(E)** In Case 4, the serial structure of the model has been removed by targeting afferent input to all the regions, that is, to the core, the belt, and the parabelt. The intracortical connections in this case were left unchanged, resulting in a connection matrix similar to the intact version of the model.

To study how temporal binding emerges out of the model, three experiments were carried out in which (1) model structure, (2) adaptation decay time, and (3) the proportion of functionally inhibitory *W*_ie_ connections were varied in turn. In each experiment, the effect of these variations on two measures were quantified: first, the proportion of *spectrally selective* columns for which *PI* < 3; second, the proportion of *temporal CS* columns, that is, those whose responses required temporal binding. As shown in Figure 4, the structure of the original, intact model (Case 0, Figure 4A) was varied in the first experiment by manipulating the tonotopic mappings, the feedback connectivity, the parallel stucture, and the serial structure of the model. This resulted in four modified versions of the model: in Case 1 (Figure 4B), the topographic connectivity between areas was transformed into random connectivity. In Case 2 (Figure 4C), the parabelt-belt and belt-core feedback connections were removed. In Case 3 (Figure 4D), the parallel structure was removed by redistributing the connections so that all areas became interconnected with equal connection density. To maintain the serial structure, no interconnections between the core and parabelt were allowed in this case. Finally, in Case 4 (Figure 4E), the serial structure was removed by having afferent connections target not only the three core areas, but also the belt and parabelt areas. In these modifications, the intra-area connectivity, including the inhibitory connections, were left intact. Thus, the global proportion of inhibitory connections remained at the default (Case 0) level of around 50% in Cases 1, 3, and 4. In Case 2, where the excitatory feedback connections were cut, the proportion of inhibitory connections rose to 56%. In the second experiment, the intact model was again used, but the adaptation time constant was manipulated in five logarithmic steps in the 50–800 ms range. In the third experiment, the proportion of functionally inhibitory connections (i.e., those described in the inter-neuron targeting *W*_ie)_ was varied by changing *p*_inh_ from zero to one in 10 steps.

The various model structures were quantified by using the scaled small-world index σ (Watts and Strogatz, 1998; Rubinov and Sporns, 2010). Small-worldness seems to be a characteristic of cortical connectivity and is thought to contribute to the efficiency of cortical functioning (Hilgetag and Kaiser, 2004; Sporns and Zwi, 2004; Sheppard et al., 2012). Small-world networks inhabit the continuum between regular architectures and random networks, and are characterized by node clusters and by all nodes being within close reach of each other in terms of node-to-node distance. That is, unlike regular networks, small-world networks have short average node-to-node path lengths λ and, unlike random networks, they have a high clustering coefficient γ which expresses the probability that connection neighbors of a node are also connected to each other. The index σ is the ratio γ/λ with values >1 indicating a small-world structure (both γ and λ are scaled relative to equivalent values in random networks). Here, the small world index was calculated for the total directed connectivity between columns, irrespective of whether the connections were functionally excitatory or inhibitory. Thus, the analysis targeted a combination of the weight matrices *W*_ee_ and *W*_ie_.

To further explore the effect of small-world connectivity on the emergence of stimulus and combination selectivity, we varied the structure of a 208 node network which was identical to the intact model of auditory cortex except for its inter-column connectivity. That is, *W*_ei_ was left untouched as were the diagonal values of *W*_ee_ and *W*_ie_. To gain the off-diagonal terms of *W*_ee_ and *W*_ie_, a single 208 × 208 matrix *L* was first constructed and manipulated in terms of its small-world index σ. At the regular network extreme, columns were connected to each other in a nearest neighbor fashion within 10 column distance: *L*_ij_ > 0 iff |i − j |≤ 10, 0 otherwise. This resulted in a network with *nz* = 4466 connections. To gain variation in the small-world index of the network, *n*_rw_ connections between random column pairs where reassigned to other pairs. The probability *p*_rw_ of this rewiring was varied from 0 to 1. As a result, the small-world index increased from an initial σ = 2 at *p*_rw_ = 0 to σ = 5 at *p*_rw_ = 0.1. As *p*_rw_ was further increased to *p*_rw_ = 1, σ decreased monotonically to σ = 1. For each instance of *p*_rw_, the matrix *L* was separated into the diagonal terms of *W*_ee_ and *W*_ie_. To keep the proportion of functionally inhibitory connections the same as in the intact model, 50% of *L* connections were randomly assigned to *W*_ee_ (with a magnitude of 0.5) and 50% to *W*_ie_ (magnitude 2.0).

Because of the stochastic nature of the synaptic weight distributions, each variation of the model was generated 30 times. These sets provided the mean measurement values and standard errrors of the mean (sem) reported in the Results section. In each of the three experiments, a set of 30 models with the default settings was generated anew to provide a built-in replication of the performance of the original model. Repeated-measures analysis of variance (ANOVA) was used to analyze the effects of structure, adaptation time constant, proportion of inhibitory connections and region (i.e., core, belt, parabelt) on (1) the proportion of spectrally selective columns and (2) the proportion of columns exhibiting temporal binding. Newman–Keuls *post-hoc* tests were performed when appropriate. In addition, the effect of the small-world index σ on the number of spectrally selective and temporally sensitive columns in a random network were inspected via regression analysis.

## Results

**Figure 5:**
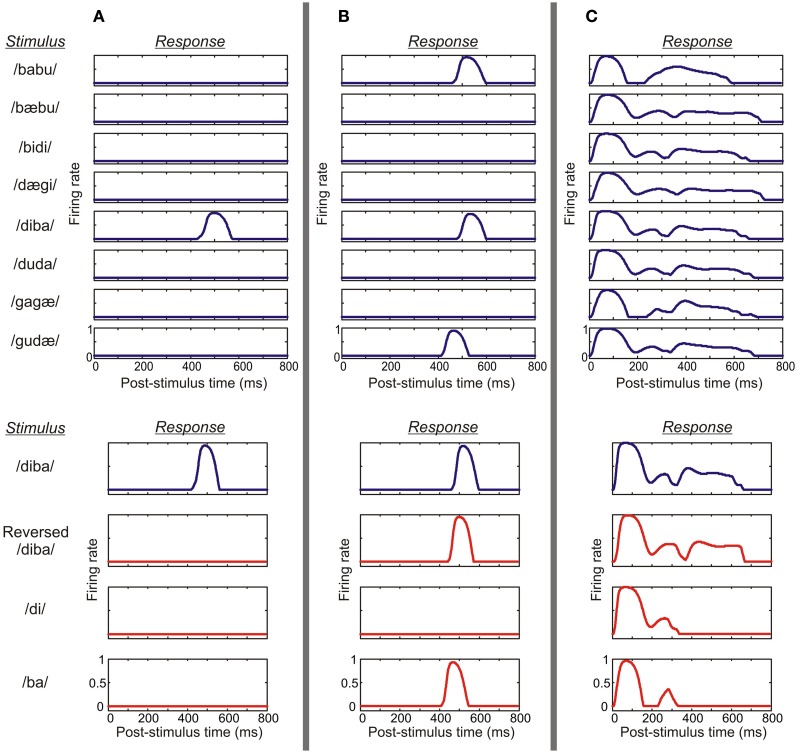
**A demonstration of various response profiles from three columns. (A)** This example shows the activity of the pyramidal cell population of a parabelt column which displayed both spectral selectivity and temporal binding. Top: the column responded to the stimulus /diba/ at a latency of around 500 ms, but generated no response to any of the other seven pseudowords. Thus, the column was spectrally selective, showing strong preference with *PI* = 1. Bottom: the column responded to its preferred stimulus (blue curve), but failed to respond to the time-reversed version of this pseudoword or to the CV constituents presented in isolation (red curves). Thus, the column showed temporal CS indicating the presence of temporal binding. **(B)** An example from the belt shows a column which again is spectrally selective, responding to a subset of three pseudowords (*PI* = 3). However, in this case, the column responds not only to the preferred stimulus/diba/ but also to the time-reversed version of this pseudoword and to the CV syllable/ba/. Thus, the activity of the column does not indicate temporal binding. **(C)** In this example, the column shows weak preference, responding equally strongly to all eight pseudowords (*PI* = 8). Also, strong responses are elicited by reversed pseudowords and the CV constituents. Thus, the column was neither spectrally selective nor showed temporal binding.

A set of eight CVCV pseudowords were used as stimulus material. The behavior of the model was quantified by examining the firing rates of the excitatory (pyramidal) cell populations of each microcolumn. With the default parameter values, the microcolumns of the model exhibited a variety of response profiles, as shown in Figure 5. A majority of the columns were strongly selective to the pseudowords (Figures 5A,B), that is, they responded to only three or fewer of the stimuli (*PI* ≤ 3; *N* = 122 ± 2 sem out of 208). A minority of the columns showed temporal CS (CS; *N* = 39 ± 3): they responded prominently to their PS but, importantly, produced either weak or no responses to the isolated CV elements of the PS and to its time-reversed version (Figure 5A). This was in contrast to columns which were selective to particular stimuli (i.e., *PI* ≤ 3) but which did not fulfill the requirements of CS (*N* = 86 ± 2; Figure 5B), as well as to columns which were not selective to the stimuli (*PI* > 3; *N* = 86 ± 2; Figure 5C). The scaled small-world index had a value of σ = 3.2 indicating that the model was of the small-world type.

Inspecting the model behavior at a finer resolution, spectral selectivity was evident in all regions: In the core, 71% (±1% sem) of columns exhibited strong preference, that is, they had a PI in the 1–3 range. In the belt and parabelt, strong preference was evident in 55% (±1%) and 56% (±1%) of columns, respectively. Columns exhibiting temporal CS were also present in all regions. However, they were relatively rare in the core region (*P* = 5 ± 1%) but more frequently found in the belt (*P* = 23 ± 2%) and the parabelt (*P* = 24 ± 2%).

**Figure 6:**
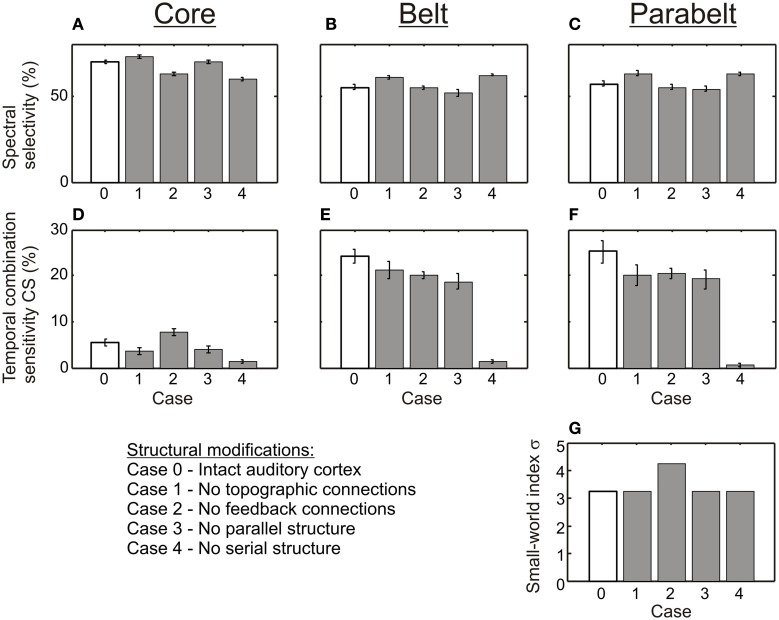
**The effect of structure on spectral selectivity and temporal binding. (A–C)** The proportion of columns exhibiting spectral selectivity (*PI* ≤ 3) was weakly affected by the structural modifications (shown in Figure 4). In all modifications (Cases 1–4, gray bars), spectral selectivity in the core **(A)**, the belt **(B)**, and the parabelt **(C)** region remained at the high level found in the intact version of the model (Case 0, white bars). **(D–F)** In contrast, temporal binding was more sensitive to structural changes, as was evident in the core **(D)**, the belt **(E)**, and the parabelt **(F)**. In most modifications (Cases 1–3), the proportion of CS columns remained at the same magnitude level as in the intact model (Case 0). However, when the serial structure was removed, very few CS columns could be found in any region. Thus, a serial structure seems to be necessary for temporal binding. **(G)** The small world index σ remained high for all versions of the model. Error bars indicate sem.

As shown in Figure 6, the variations in the structure of the model had differential effects on spectral selectivity and temporal CS. The proportion of spectrally selective columns (*PI* ≤ 3) varied slightly according to structure [*F*_(4, 116)_ = 25.3, *p* < 0.001] and this variation depended on region [*F*_(8, 232) = 14.2_, *p* < 0.001]: In the core region, this proportion remained in the 60–70% range for all structural modifications, and in the belt and parabelt it remained in the 50–60% range. In contrast, temporal binding showed a more complex dependence on structure [*F*_(3, 116)_ = 54.5, *p* < 0.001; region-structure interaction *F*_(8, 232)_ = 27.4, *p* < 0.001]. When inter-area topographic connections were randomized (Case 1), feedback connections were abolished (Case 2), or when the parallel structure was removed (Case 3), the proportion of CS columns in the belt and parabelt shrank from around 25% to around 20% (belt: Case 0 vs. Case 3 *p* < 0.05, *p* = n.s. for other comparisons to Case 0; parabelt: *p* < 0.01 for all comparisons to Case 0). In the core, this proportion remained below 10% in all cases. Importantly, when the serial structure of the model was corrupted by having afferent input target not only the core but the belt and parabelt also (Case 4), the proportion of CS columns drastically shrank to below 2% in the core, the belt, and the parabelt region (core: *p* < 0.05; belt and parabelt: *p* < 0.001 in all comparisons to Case 0). None of the structural variations affected the small-world nature of the network, with σ > 3 in all cases. Overall, the proportion of CS columns was larger in the belt and parabelt than in the core [*F*_(2, 58)_ = 165.1, *p* < 0.001].

**Figure 7:**
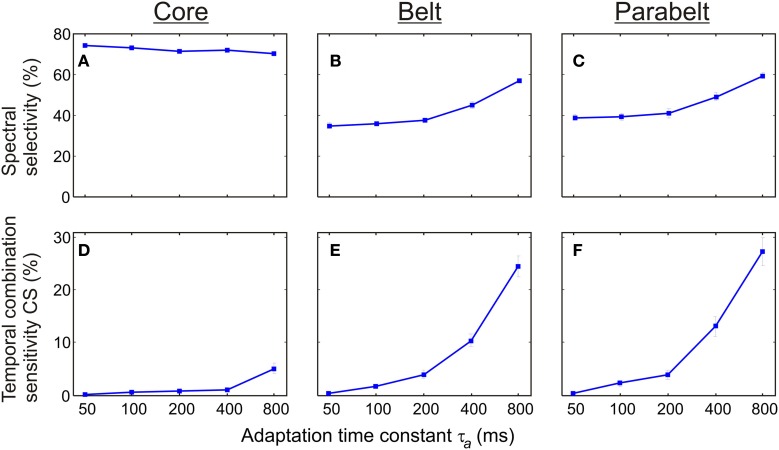
**The effect of synaptic depression (adaptation) on spectral selectivity and temporal binding. (A–C)** The proportion of columns showing spectral selectivity (*PI* ≤ 3) was differentially sensitive to the adaptation time constant τ_a_ according to cortical region. In the core **(A)**, this proportion remained above 70% for all values of τ_a_ In the belt **(B)** and parabelt **(C)** the frequency of spectrally selective columns increased from 40 to 60%. **(D,E)** In all regions, the proportion of temporal CS columns was an increasing function of the adaptation time constant. With τ_a_ = 50 ms, no such columns could be detected in the core **(D)**, the belt **(E)**, or the parabelt **(F)**. When τ_a_ was increased to 800 ms, CS columns constituted 5% of the core and around 25% of the belt and parabelt. Thus, adaptation contributes to both spectral selectivity and temporal binding. Error bars indicate sem.

As shown in Figure 7, the proportion of spectrally selective columns increased as a function of the adaptation time constant τ_a_ [*F*_(4, 116)_ = 34.4, *p* < 0.001] although there were differential effects of τ_a_ in the core, belt, and parabelt region [*F*_(8, 232)_ = 21.4, *p* < 0.001]. An increase of τ_a_ from 50 to 800 ms had little impact on spectral selectivity in the core region, where the proportion of columns with *PI* ≤ 3 remained at around 70% (*p* = n.s. for all *post-hoc* comparisons). In contrast, spectral selectivity increased as a function of τ_a_ in both the belt and the parabelt region: the proportion of spectrally selective columns rose from 35 to 57% in the belt region (*p* < 0.001), and from 39 to 59% in the parabelt (*p* < 0.001). Also, temporal CS exhibited dependence on adaptation [*F*_(4, 116)_ = 63.7, *p* < 0.001] which was differentially affected by region [*F*_(8, 232)_ = 43.2, *p* < 0.001]. In the core region, very few columns with CS were detected when τ_a_ was below 400 ms (*P* < 1%), and emerged only when τ_a_ was increased to 800 ms (*P* = 5%). In the belt, the proportion of CS columns monotonically increased as a function of τ_a_ from 0 to around 24%. A similar effect was observed in the parabelt, where CS columns increased from 0 to 27%. For comparison, we also ran a set of simulations, where the adaptation had been removed (i.e., *a*_ij_ = 1, ∀*i*, ∀*j*). In this situation, no CS columns were found in the model. In general, compared to the belt and parabelt, the core region contained a larger number of spectrally selective columns [*F*_(2, 58)_ = 1228.0, *p* < 0.001] and a smaller number of columns exhibiting temporal binding [*F*_(2, 58)_ = 166.3.0, *p* < 0.001].

**Figure 8:**
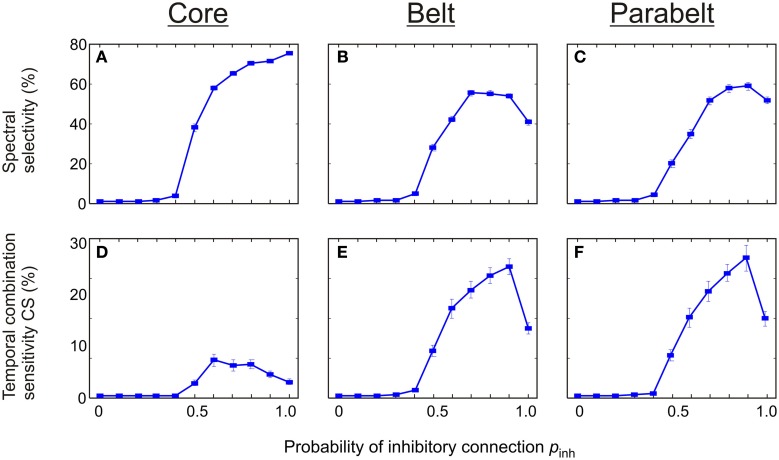
**The effect of functionally inhibitory connections on spectral selectivity and temporal binding. (A–C)** The number of spectrally selective columns increased as a function of the probability *p*_inh_ of inhibitory connections made between columns within the same area. In the core **(A)**, the belt **(B)**, and the parabelt **(C)** region, no spectral selectivity was found for *p*_inh_ < 0.4. For larger values of *p*_inh_, a maximum of around 80% was reached in the core, and 60% in the belt and parabelt. **(D–F)** Temporal binding depended on *p*_inh_ in all regions, and was missing for *p*_inh_ < 0.4. In the core **(D)**, the proportion of temporal CS columns reached a maximum of 8% at *p*_inh_ = 0.6. In the belt **(E)** and parabelt **(F)**, maxima of around 27% occurred when *p*_inh_ = 0.9. Error bars indicate sem.

There was a strong dependence of both spectral selectivity and temporal CS on the probability of functionally inhibitory connections *p*_inh_ in the model (Figure 8). In general, the number of spectrally selective columns was an increasing function of *p*_inh_ [*F*_(7, 203)_ = 589.2, *p* < 0.001]. When *p*_inh_ was below 0.4, the model contained no spectrally selective columns. As *p*_inh_ was increased to unity, the proportion of these columns increased monotonically to around 80% in the core. In the belt, the number of spectrally selective columns increased and reached a peak of around 60% at *p*_inh_ = 0.7. Similarly, in the parabelt, a peak of around 60% was reached at *p*_inh_ = 0.9. The proportion of CS columns exhibited clear unimodal behavior [*F*_(5, 145)_ = 15.5, *p* < 0.001]: for values below *p*_inh_ = 0.4, there were no CS columns. As *p*_inh_ was increased beyond 0.4, the proportion of these columns in the core reached a maximum of around 10% at *p*_inh_ = 0.6. In the belt and parabelt, a maximum of around 28% occured when *p*_inh_ = 0.9.

**Figure 9:**
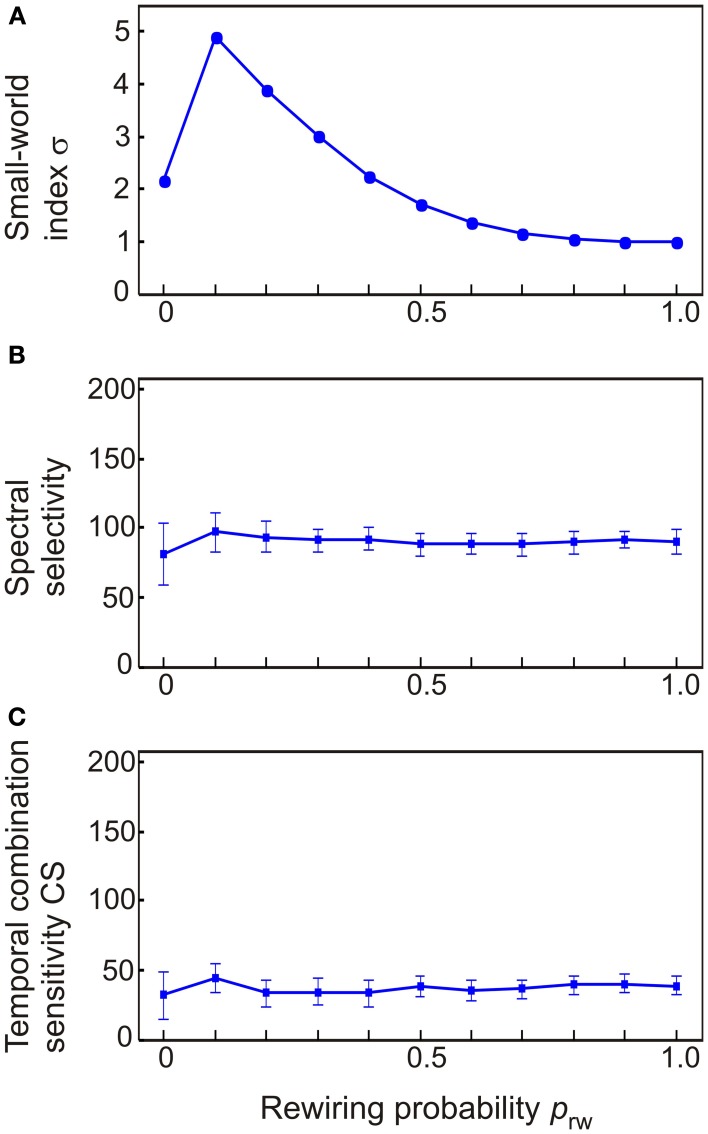
**The dependence of spectral selectivity and temporal binding on the small-world index σ. (A)** Variations in the small-world index were generated by starting off with a regular network where columns were connected to only their nearest neighbors and then rewiring column pairs randomly with the probability *p*_rw_. This resulted in networks which either had a clear small-world structure (σ = 5) or were random (σ ≈ 1). **(B,C)** Neither spectral selectivity nor temporal binding were affected by the small-world index. Measurements are given as total number of columns out of 208. Error bars indicate sem.

To explore how stimulus and combination selectivity are affected by small-world connectivity, we varied the structure of a 208-node network which was identical to the intact model of auditory cortex except for its inter-column connectivity. In effect, the off-diagonal (lateral) terms of *W*_ee_ and *W*_ie_ were combined and redistributed into a connectivity matrix *L*. At the regular network extreme, columns were connected to each other in a nearest neighbor fashion (reflected in *L* having non-zero values along a diagonal stripe). To gain variation in σ, connections between random column pairs where reassigned to other pairs. The probability *p*_rw_ of this rewiring was varied from 0 to 1 (at *p*_rw_ = 1, non-zero values of *L* were randomly distributed). As shown in Figure 9A, the small-world index σ increased from an initial σ = 2 to σ = 5 at *p*_rw_ = 0.1; the index σ decreased monotonically to σ = 1 as *p*_rw_ was further increased to *p*_rw_ = 1. The small world index correlated with neither the number of spectrally selectivity columns [*R*^2^ = 0.03, *F*_(1.3)_ = 11.1, *p* < 0.001; Figure 9B] nor the number of CS columns [*R*^2^ = 0.001, *F*_(1.3)_ = 0.4, *p* = n.s.; Figure 9C].

## Discussion

The binding of spectral information over time represents a computational challenge which the auditory cortex seems to be especially adept at dealing with. As the underlying mechanisms allowing this have remained obscure, the current study explored how the structure and dynamics of auditory cortex contribute to the ability of the brain to represent sounds characterized by a complex, time-evolving structure. We constructed a model of interacting cortical microcolumns with a connectivity pattern mimicking the core-belt-parabelt structure found in the auditory cortices of humans and non-human primates (Figure 2). This organization entails the presence of several parallel streams of feedforward activation progressing in a serial manner from the core to the parabelt (for reviews, see Pandya, 1995; Kaas and Hackett, 2000; Eggermont and Ponton, 2002). Further, the excitatory synapses between pyramidal cells exhibited activity-dependent depression (adaptation). This adaptation depended on past pre-synaptic stimulation and evolved with a relatively long time constant of 800 ms (see May and Tiitinen, 2010). The model showed selectivity to both the spectral and temporal aspects of stimulation, and thus allowed us to probe further what the contributing factors to temporal binding might be.

When the model was presented with a selection of eight CVCV pseudowords, each having a unique spectral composition, a majority of the columns (122 out of 208) were found to be selective to the stimuli in the sense that each responded only to a small stimulus subset (*PI* ≤ 3). This selectivity resembles that found in primate auditory cortex for species-specific communication sounds (Rauschecker et al., 1995; Wang et al., 1995; Rauschecker, 1997; Tian et al., 2001; Recanzone, 2008). While this result implies that the columns of the model are able to perform spectral segregation of stimuli, this selectivity in itself does not yet entail that the columns were performing *temporal* binding of spectral information. To probe for temporal binding we used time-reversed versions of the pseudowords as well as the CV constituents of the words, presented in isolation (Figure 3). These modified stimuli were thus spectrally identical to the original stimulus set, but either lacked the original temporal structure or amounted to presenting parts of the original stimulus without the correct historical context. Therefore, responses to the original, intact stimulus combined with weak responses to the modified stimuli implies combination selectivity (CS), that is, selectivity to the specific ordering of the spectral information in time. The simulations showed that a number of columns (39 out of 208) exhibited CS, and these were mostly found in the belt and parabelt regions. In summary, the model seems to be able to both segregate the stimuli according to spectral structure and to perform temporal binding of this structure. The model predicts that spectral selectivity is a common feature of auditory cortex, found in 59% of columns, in the core, belt, and parabelt regions. In contrast, temporal binding is evident in a far smaller proportion of columns (19%), which are mostly located in the belt and parabelt.

Our simulations are in line with the intracortical results of Recanzone (2008), who inspected the temporal binding ability of cells in several areas belonging to either the core or belt region of the auditory cortex of the macaque monkey. Using monkey calls and their time-reversed counterparts, he found that only 10–20% of cells exhibited temporal CS. While this agrees with our results, Recanzone found no difference between the core and belt areas in terms of CS. This discrepancy with our results might be due to the default settings of the current model. For example, by decreasing the probability of intra-region inhibitory connections from 0.8 to 0.6, thereby reducing the global proportion of inhibitory connections from 50 to 37%, the difference between the frequency of CS columns in the core and belt is reduced (see Figures 8D–F). However, it must be emphasized that Recanzone presented alert animal subjects with behaviorally significant stimuli. In contrast, the simulations of the current study were designed to address the basic mechanisms of temporal binding, and as such did not include top–down effects mediating stimulus significance and attention.

The original, intact version of the model incorporated several features of auditory cortex including: (1) topographic connectivity between areas, (2) the feedback connections from parabelt to belt and from belt to core, (3) the presence of multiple parallel core-belt-parabelt streams, (4) the serial core-belt-parabelt structure in itself. When these features were corrupted each in turn (Figure 4), spectral selectivity remained intact (Figure 6). In contrast, temporal binding appeared to be more sensitive to structural variations. In the core, the proportion of columns with temporal CS was little affected by the absence of topographic connections, feedback connections, or the presence of multiple parallel streams. In the belt and parabelt, there was a small decrease in this proportion (from 25 to 20%). In stark contrast, when the serial structure of the model was abolished by adding afferent input to the belt and parabelt, the proportion of CS columns collapsed to a negligible level (<2%) in all regions of the model. It would be interesting to see whether this sensitivity applies also in the case of other manipulations in serial strucure, for example if the core areas were directly connected with the parabelt. It is surprising that feedback connectivity had a relatively small effect on model performance, given that feedback connections from cortex to subcortical areas have a strong effect on the tuning properties of subcortical neurons (Suga, 2012) and their temporal window of integration (Balaguer-Ballester et al., 2009). The current results tentatively suggest that cortico-cortical feedback does not participate in spectral and temporal processing as such but that its functional significance lies elsewhere, perhaps in mediating attentional effects and the task relevance of the stimuli. In addition, a potentially interesting aspect of the model was its small-world structure, the intact version having a scaled small-world index value of 3.2. Therefore it was characterized both by clustering and short column-to-column path lengths. However, simulations using random networks revealed that small-worldness in itself contributed to neither spectral selectivity nor temporal binding (Figure 9). In sum, while the ability of the model to perform spectral and temporal segregation was robust in the face of structural modifications, it seems that the serial structure of auditory cortex is a necessary condition for temporal binding to occur.

We varied the time constant of adaptation τ_a_ in the 50–800 ms range and found that the number of CS columns increases monotonically as a function of τ*_a_* (Figure 7). Indeed, shortening the decay time to 50 ms completely abolished the temporal binding found in the model. The maximum value of τ_a_ (800 ms) was slightly larger than the average stimulus duration (660 ms). A question for further investigation is therefore whether optimum performance in terms of the number CS columns depends on the ratio between stimulus duration and the adaptation time constant. For example, if τ_a_ were further increased, would the number of CS columns keep growing, or would it plateau out or even start shrinking? The spectral selectivity to the stimuli fared better at the reduction of adaptation decay time. In the core region, selectivity remained at or above 70% for all values of τ_a_. In the belt and parabelt, selectivity was reduced from around 60% to around 40% as τ_a_ sank from the default value of 800 ms to 50 ms. Thus, temporal binding was more sensitive than spectral selectivity to variations in the adaptation decay time. Importantly, these results imply that adaptation with a long decay constant—matching the time span of the stimuli—could provide the central mechanism through which cells in auditory cortex become sensitive to the temporal structure of sound. These results imply that stimulus-specific adaptation which is observed both intracortically (Ulanovsky et al., 2004) and non-invasively (May and Tiitinen, 2010) not only leads to diminished responses when stimuli are repeated in laboratory conditions, but actually plays a central role when the brain forms representations of naturally occurring sounds with a rich spectral and temporal structure.

Inhibition was assumed to be local in the sense that functionally inhibitory connections (i.e., interneuron-targeting excitatory connections) to a column always originated from the area of origin of that column. The global proportion of functionally inhibitory connections in the model was around 50%. While intra-area inhibition has been established (Kurt et al., 2008; Moeller et al., 2010), there is no experimental justification for the lack of long-range inhibitory connections or for the 50% proportion of inhibitory connections used in the model. Indeed, as discussed above, this estimate may be too high in view of the results of Recanzone (2008). With these considerations, additional simulations were run in which inhibition was switched from local (intra-area) to global. Except for increasing somewhat the proportion of CS columns in the core, these produced no change in model performance and are not shown in this study. We also parametrically varied the propensity *p*_inh_ of inhibitory connections within each area, and found that this had a clear effect (Figure 8). Both spectrally selective columns and those showing temporal CS were negligible in number when the probability of inhibitory connections was below 0.5. As *p*_inh_ was increased, the proportion of selective columns increased and peaked at around 28% when *p*_inh_ = 0.9. Thus, the presence of inhibitory connections between columns appears to be crucial for both spectral selectivity and for temporal binding.

Our simulations replicate the results from the primate brain whereby cells in the anterior auditory and prefrontal cortex respond selectively to vocalizations (Rauschecker et al., 1995; Tian et al., 2001). Given that most neurons prefer more than one stimulus (both experimentally and in simulations), it is unlikely that speech sounds are represented via “grandmother cells” in a place code. It is more likely that a population code is used, and one might speculate that this exhibits the robustness necessary for speech recognition to occur in noisy environments—one of the primary and most difficult challenges in current research on automatic speech recognition (Scharenborg, 2007). Therefore, an explanation of how the brain identifies speech sounds despite a great variation in their acoustic structure, might benefit from the model of temporal binding presented here. This model together with methods employed in May and Tiitinen (2010) could, hypothetically, be tested with recent MEG results showing how human cortex is activated by noisy speech sounds (Miettinen et al., 2010, 2011, 2012; Tiitinen et al., 2012).

It should be emphasized that temporal binding and spectral selectivity to speech stimuli in the current model was not the result of training, that is, long-term adaptation to the statistical structure of the stimulus environment. Rather, selectivity was instantaneously available, the presence of a serial structure coupled with synaptic depression and inter-column inhibition being sufficient conditions for it to emerge. In this aspect, the current model fits into the general framework of state-dependent computations (also known as reservoir computing, echo-state networks, and liquid-state machines) which are a promising new approach for teaching artificial neural networks to make non-linear and chaotic mappings (Jaeger and Haas, 2004; for a review, see Lukoševicius and Jaeger, 2009) and may be useful as models of brain function (for a review, see Buonomano and Maass, 2009). Indeed, an interesting extension of both the current model and of state-dependent computations generally may be to study how long-term exposure to a particular stimulus environment (e.g., in terms of species-specific vocalizations or a native language) affects temporal binding and the mapping of complex stimuli.

In conclusion, the current simulations suggest that spectral selectivity is a robust feature which survives a wide range of simulated parameter changes. In contrast, temporal binding seems to require tuning of the structure and dynamics of auditory cortex: It is strongly dependent on the presence of synaptic depression (adaptation) with a slow decay time arching over at least the time span of the stimuli. Adaptation modifies the interaction weights between microcolumns in a stimulus-specific way. Thus, the input-output mapping of auditory cortex—occurring on the fast time scale of firing rate changes—is not static but, rather, depends dynamically on the immediate stimulus history. Temporal binding can therefore be seen to emerge out of the presence of slow and fast dynamics in the auditory cortex neural network. The exact connection of adaptation time spans and stimulus length remains an interesting question which could be approached by using stimuli with a larger variation in duration than that of the current stimulus set. Further, the serial structure of auditory cortex whereby feedforward activation progresses in an orderly manner through several processing stages appears to be necessary for the ability of cells in the belt and parabelt to perform temporal binding. This was evident not only in simulations where the serial structure was tampered with but also when looking at the simulations as a whole. Compared to the core region, the belt and parabelt—which were at least one synaptic connection further away from afferent input than the core—exhibited temporal binding more often and this ability was more sensitive to parameter changes. Thus, it seems that the serial structure contributes to temporal binding by “isolating” the downstream parts of the auditory system from stimulus-following afferent input, thereby creating a subsystem (i.e., the belt and parabelt) where all excitatory inputs to pyramidal cells arrive via adaptive synapses. These observations might partly explain why structural seriality seems to be a peculiar feature of auditory cortex (Kaas and Hackett, 2000): as the temporal dimension of stimulation is the defining feature of auditory information, a system which has evolved to process sound will probably utilize structural solutions which support the emergence of temporal binding.

### Conflict of interest statement

The authors declare that the research was conducted in the absence of any commercial or financial relationships that could be construed as a potential conflict of interest.
